# GPR110 (ADGRF1) mediates anti-inflammatory effects of *N*-docosahexaenoylethanolamine

**DOI:** 10.1186/s12974-019-1621-2

**Published:** 2019-11-15

**Authors:** Taeyeop Park, Huazhen Chen, Hee-Yong Kim

**Affiliations:** Laboratory of Molecular Signaling, National Institute of Alcohol Abuse and Alcoholism, 5625 Fishers Lane, Rm. 3N-07, Rockville, MD 20852 USA

**Keywords:** GPR110, Synaptamide, cAMP, Neuroinflammation, NF-κB, Docosahexaenoic acid, Cytokine

## Abstract

**Background:**

Neuroinflammation is a widely accepted underlying condition for various pathological processes in the brain. In a recent study, synaptamide, an endogenous metabolite derived from docosahexaenoic acid (DHA, 22:6n-3), was identified as a specific ligand to orphan adhesion G-protein-coupled receptor 110 (GPR110, ADGRF1). Synaptamide has been shown to suppress lipopolysaccharide (LPS)-induced neuroinflammation in mice, but involvement of GPR110 in this process has not been established. In this study, we investigated the possible immune regulatory role of GPR110 in mediating the anti-neuroinflammatory effects of synaptamide under a systemic inflammatory condition.

**Methods:**

For in vitro studies, we assessed the role of GPR110 in synaptamide effects on LPS-induced inflammatory responses in adult primary mouse microglia, immortalized murine microglial cells (BV2), primary neutrophil, and peritoneal macrophage by using quantitative PCR (qPCR) and enzyme-linked immunosorbent assay (ELISA) as well as neutrophil migration and ROS production assays. To evaluate in vivo effects, wild-type (WT) and GPR110 knock-out (KO) mice were injected with LPS intraperitoneally (i.p.) or TNF intravenously (i.v.) followed by synaptamide (i.p.), and expression of proinflammatory mediators was measured by qPCR, ELISA, and western blot analysis. Activated microglia in the brain and NF-kB activation in cells were examined microscopically after immunostaining for Iba-1 and RelA, respectively.

**Results:**

Intraperitoneal (i.p.) administration of LPS increased TNF and IL-1β in the blood and induced pro-inflammatory cytokine expression in the brain. Subsequent i.p. injection of the GPR110 ligand synaptamide significantly reduced LPS-induced inflammatory responses in wild-type (WT) but not in GPR110 knock-out (KO) mice. In cultured microglia, synaptamide increased cAMP and inhibited LPS-induced proinflammatory cytokine expression by inhibiting the translocation of NF-κB subunit RelA into the nucleus. These effects were abolished by blocking synaptamide binding to GPR110 using an *N*-terminal targeting antibody. GPR110 expression was found to be high in neutrophils and macrophages where synaptamide also caused a GPR110-dependent increase in cAMP and inhibition of LPS-induced pro-inflammatory mediator expression. Intravenous injection of TNF, a pro-inflammatory cytokine that increases in the circulation after LPS treatment, elicited inflammatory responses in the brain which were dampened by the subsequent injection (i.p.) of synaptamide in a GPR110-dependent manner.

**Conclusion:**

Our study demonstrates the immune-regulatory function of GPR110 in both brain and periphery, collectively contributing to the anti-neuroinflammatory effects of synaptamide under a systemic inflammatory condition. We suggest GPR110 activation as a novel therapeutic strategy to ameliorate inflammation in the brain as well as periphery.

## Introduction

Neuroinflammation is an inflammatory condition in the central nervous system (CNS). Inflammation is initiated by various causes including infection, injury, or the exposure to toxins, often followed by the glial activation and production of inflammatory mediators. Growing evidence identifies neuroinflammation as an underlying condition or even a cause for the progression of neurodegenerative diseases such as Alzheimer’s disease, Parkinson’s disease, and multiple sclerosis [1–4].

Microglia, the resident immune cells of the central nervous system (CNS), have long been considered the primary player for neuroinflammation [5, 6]. Nevertheless, inflammatory cytokines IL-1α, IL-6, and TNF have been shown to cross the blood-brain barrier (BBB) through saturable transport systems [7–9]. It has been also reported that TNF receptors at the BBB function as its transporters [10, 11], and systemic TNF administration leads to microglia activation and dopaminergic neuron loss [2], suggesting active participation of the peripheral immune system in the neuroinflammation process.

Adhesion G-protein-coupled receptors (aGPCRs) are found in most cell types, including immune cells [12]. Several aGPCR have been shown to regulate immune responses such as inflammatory gene transcription, immune cell migration, phagocyte activation, and superoxide generation through their specific G-protein subunits [13–15]. Recently, an aGPCR GPR110 (ADGRF1) was identified as the target receptor of a DHA metabolite *N*-docosahexaenoylethanolamine (synaptamide), mediating the potent neurogenic, neuritogenic, and synaptogenic activities of synaptamide in developing neurons, through activating Gα_s_-mediated cyclic adenosine monophosphate (cAMP)/PKA signaling [16, 17]. Synaptamide was also shown to inhibit LPS-induced neuroinflammation through cAMP-dependent signaling in adult mice [18], but involvement of GPR110 has not been established. In the present study, we investigated the mechanism through which synaptamide inhibits LPS-induced neuroinflammation using both in vivo and in vitro models.

## Material and methods

### Chemicals and antibodies

Dulbecco’s modified Eagle medium (DMEM) was purchased from American Type Culture Collection (ATCC, Manassas, VA). Fetal bovine serum and penicillin/streptomycin were purchased from Invitrogen (Carlsbad, CA, USA). SQ 22536 was purchased from R & D Systems, Inc (Minneapolis, MN). Lipopolysaccharides (*Escherichia coli 055:B5*) and forskolin were purchased from Sigma-Aldrich (St. Louis, MO). Anti-Iba-1 antibody was purchased from Wako (Richmond, VA, USA). Recombinant mouse TNF was purchased from BioLegend (San Diego, CA). LTB4 and mouse recombinant CCL2 were purchased from Cayman Chemical (Ann Arbor, MI) and BioLegend. GPR110 N-terminal blocking antibody was purchased from Abmart (Shanghai, China). ROS permeable indicator, Carboxy-H2DCFDA, was purchased from Invitrogen (Carlsbad, CA).

### Animals

C57BL/6J mice were purchased from Charles River Laboratories (Portage, MI, USA), and GPR110 (*Adgrf1*) heterozygous mice on C57BL/6 background were generated by the Knockout Mouse Project (KOMP) Repository (MMRRC_046507-UCD). GPR110 wild-type (WT) and knock-out (KO) mice were generated by heterozygote mating in our laboratory. All experiments in this study were carried out in accordance with the guiding principles for the care and use of animals approved by the National Institute on Alcohol Abuse and Alcoholism (LMS-HK-13). Mice 8–12 weeks of age received the intraperitoneal (i.p.) injection of 1.0 mg/kg LPS (*E. coli*, serotype 055:B5, Sigma) or intravenous (i.v.) injection of 250 μg/kg TNF (Biolegend) followed by synaptamide injection (5 mg/kg, i.p.). At desired time point, mice were deeply anesthetized with isoflurane and perfused quickly with chilled PBS for RNA isolation and western blot analysis, or chilled PBS containing 4% paraformaldehyde for immunostaining. Blood from mice were collected by cardiac puncture before perfusing with chilled PBS. For some in vitro studies using microglia, neutrophils, and macrophages, we used commercially available *C57BL/6J* male mice. However, for all experiments with matching WT and KO groups, we used both male and female mice, which were generated in house by heterozygote mating. In such case, each experimental group was assigned with approximately the same ratio of male and female mice.

### Microglia cell culture

BV2 cells, a mouse microglial cell line that was a kind gift from Dr. Ronald Mason (NIEHS, NIH), were cultured in Dulbecco’s modified Eagle’s medium (DMEM) (ATCC) containing 10% fetal bovine serum (GIBCO) and 1% penicillin/streptomycin (Invitrogen). Murine microglia or BV2 cells were treated with LPS (Sigma-Aldrich) at a concentration of 100 ng/mL with or without synaptamide at indicated concentrations. The murine primary microglial cells were isolated from non-stimulated normal brains of WT and GPR110 KO mice at age 8–10 weeks by magnetic separation. After mice were transcardially perfused with ice-cold PBS under anesthesia, brains were collected, washed with cold PBS, and cut into 8 sagittal slices which were transferred to the C tube containing enzyme mixture and dissociated with gentleMACS dissociator according to the manufacturer’s instruction (Miltenyi Biotec). Dissociated brain cells were filtered by a MACS SmartStrainer (70 μm), centrifuged at 300×*g* for 10 min, and debris were removed using the manufacturer’s removal solution (1 brain: 900 μL removal solution). The cell pellet was suspended in 90 μL PB buffer (PBS containing 0.5% bovine serum albumin), incubated with 10 μL of CD11b microbeads per 10^7^ total cells for 15 min in the dark at 4 °C, washed with 1 mL of the cold PB buffer and centrifuged at 300×*g* for 5 min. The cell-bead pellet was collected and resuspended in 500 μL of the PB buffer and applied onto the LS column (Miltenyi Biotec) which was prepared by rinsing with the 3 mL of the cold PB buffer in the magnetic field. The microglia cells captured on the beads were retained on the column while non-target cells passed through the magnetic field. After removing the column from the magnetic separator, 5 mL of the PB buffer was added onto the LS column, and the microglia cells on beads were immediately flushed out by firmly pushing the plunger into the column. After centrifuging at 300×*g* for 10 min, the pellet was suspended with culture medium (DMEM containing 10% FBS, 4 mM l-glutamine and 1% penicillin/streptomycin) and plated on the poly-d-lysine-coated 12-well plates at a density of 5 × 10^5^ cells per well without bead detachment. After cells were attached on the plate by incubating overnight at 37 °C in 5% CO_2_, non-adherent cells were removed by washing three times with PBS at 12, 24, and 48 h after isolation, and treated with LPS in DMEM containing 1% FBS, 4 mM l-glutamine and 1% penicillin/streptomycin. The purity of our microglia preparation was confirmed by flow cytometry (fluorescence-activated cell sorting, FACS) after isolation using CD11b and CD45 as markers to distinguish the microglia (CD45^low^/CD11b^+^) and macrophage (CD45^high^/CD11b^+^) population (Additional file 1: Figure S1).

### Isolation of mouse neutrophil

Neutrophils were obtained from the blood of 8–10-week-old GPR110 WT and KO mice. Mice were anesthetized with isoflurane and the blood collected by cardiac puncture. After centrifugation (300×*g*/5 min at room temperature), the cells were resuspended in an appropriate volume of specific antibody cocktail and the suspension was constantly mixed in a rotating shaker at 4 °C for 10 min. The supernatant was removed by centrifugation (300×*g*/5 min), and the cell pellet was suspended in 100 μl MACS Buffer per 10^7^ total cells. After 15 μl of “Anti-Biotin-Microbeads” (Miltenyi Biotec, Bergisch Gladbach, Germany) per 10^7^ total cells were added, the suspension was incubated on the rotating shaker for 15 min. The cells then were washed with 10 ml MACS buffer (300×*g*/10 min), taken up in 500 μL of this solution and filled into a pre-equilibrated “LS MACS column” (Miltenyi Biotec). After liquid flow stopped, the column was washed three times with 3 mL MACS buffer and finally once with 5 mL MACS buffer and the entire flow-through was collected. The obtained cells were then washed once with 10 mL PBS, and in a closing step, the neutrophil cell number was assessed. The cells were resuspended in RPMI 1640 supplemented with 1% FBS and 1% penicillin/streptomycin and immediately treated for stimulation at 37 °C in 5% CO_2_. After isolation, the neutrophils were also identified by using the specific marker Ly6G by RT-PCR. Human neutrophil, peripheral blood mononuclear cells (PBMC), and platelet from healthy volunteers were isolated with an elutriator at the NIH Blood Bank. The research using blood and blood components was approved (99-CC-0168) by NIH Clinical Center for Collection and Distribution of Blood Components from Healthy Donors for In Vitro Research Use.

### Isolation of peritoneal macrophage

Mice were euthanized by CO_2_ asphyxiation, and primary macrophages were collected by peritoneal lavage of 8–10 weeks old WT or KO as well as C57/BL6J mice using 10 ml of ice-cold phosphate-buffer saline (PBS) with 2% FBS and penicillin/streptomycin. The cell pellet was suspended in macrophage culture medium containing DMEM supplemented with 10% FBS and 1% penicillin/streptomycin and incubated overnight at 37 °C in 5% CO_2_. Cultures were washed three times with PBS to remove nonadherent cells and maintained in culture medium until treatment.

### Immunostaining

Cells stimulated with LPS (100 ng/mL) were fixed with 4% paraformaldehyde for 30 min at room temperature, washed with 0.1 M Tris-buffered solution (pH 7.5, TBS), blocked with 10% normal goat serum in TBS containing 0.3% Triton X-100 at room temperature for 60 min, and incubated overnight with mouse-anti-RelA antibody (1:400, Santa Cruz) at 4 °C. The cells were washed with TBS and incubated with Alexa Fluor 488-conjugated secondary antibodies (1:1000, Life Technologies Corporation) at room temperature for 60 min. To visualize nuclei, cells were counterstained with 2 μg/mL 4′,6-diamidino-2-phenylindole (DAPI). Finally, the cells were mounted with 80% (vol/vol) glycerol, visualized under a fluorescent microscope (IX81, Olympus Corp., Tokyo, Japan), and the image data were processed using MetaMorph (Molecular Devices, Sunnyvale, CA, USA) for quantitative information. For staining brain samples, mice were anesthetized with isoflurane and transcardially perfused with 0.1 M phosphate buffer (PB, pH 7.4) containing 4% paraformaldehyde (wt/vol), and the brains were carefully removed. After overnight fixing in 4% paraformaldehyde solution followed by submerging in 30% sucrose solution at 4 °C, brains were embedded with O.C.T. compound (Tissue-Tek, 4583) medium, frozen on dry ice and stored at − 80 °C freezer until use. Coronal sections (30 μm) were prepared using Leica Cryostat and stored into the cryoprotective solution at − 20 °C. Brain sections at the approximately same position from Bregma were immunostained using anti-Iba-1 (Wako, catalogue number 019-19741) antibody followed by Alexa fluor-488-conjugated F (ab’)2 fragment goat anti-rabbit IgG (Jackson ImmunoResearch labs, catalogue number 111-546-003).

### FACS analysis

Isolated cell suspensions were incubated with a FcR blocker (5 μL/2.5 × 10^5^ cells; BD Bioscience, San Diego, CA) to reduce nonspecific antibody binding. The panel of antibodies used in these experiments included CD45-APC-Cy7 (clone 30-F11, BioLegend, San Diego, CA) and CD11b-PE (clone M1/70, Thermo, Waltham, MA). Cell suspensions were made in 350 μL of staining buffer (PBS with 2% fetal bovine serum) containing the labeled antibodies (1 μL of CD45 and CD11b) and kept on ice for 10 min. Cells were washed twice, resuspended in 1% PFA, and after 15 min, washed twice and resuspended for flow cytometry analysis using CytoFLEX flow cytometer (Beckman Coulter, Brea, CA) with FlowJo 76.1 software (TreeStar, San Carlos, CA).

### cAMP assay

Cultured microglia (2.5 × 10^5^ cells in 0.5 mL) were treated with synaptamide for 15 min, and the cAMP level was determined using a cyclicAMP XP® assay kit (Cell signaling, Danvers, MA) according to the manufacturer’s protocol. Briefly, cells were lysed using a lysis buffer including protease inhibitor cocktail (Cell Signaling), and the cell lysate was added to the cyclicAMP XP® assay kit to displace HRP-linked cAMP bound to an anti-cAMP XP® Rabbit mAb immobilized onto a 96-well plate. After removing displaced HRP-linked cAMP, the HRP substrate TMB was added and cAMP concentration was measured colorimetrically at 450 nm.

### RNA isolation and quantitative RT-PCR

Total RNA was extracted using Trizol according to the manufacturer’s protocol (Invitrogen, UK). The RNA was treated with DNase I to remove any contaminating genomic DNA (RQ1 RNase-Free DNase; Promega) and then used for cDNA synthesis applying reverse transcription reagents (Applied Biosystems Inc., Foster City, CA, USA). Expression of mRNA for TNF-α, iNOS, IL-1β, IL-6, IL-10, CCL2, and GAPDH were measured via a SYBR Green-based real-time RT-PCR assay. Samples were analyzed in triplicate on an ABI Prism 7900HT sequence detection system and QuantiTect SYBR Green PCR kit (Qiagen, CA, USA). The amplification conditions were 50 °C for 2 min, then 95 °C for 15 min, followed by 40 cycles of 95 °C for 15 s and 55 °C for 30 s. SDS 2.4 software (Applied Biosystems Inc., Foster City, CA, USA) was used to calculate the cycle threshold (Ct) in real-time assays. ΔΔCt was used to estimate the differential gene expression between samples [19]. The relative expression of mRNA was calculated after normalization to GAPDH mRNA. Primer sequences are indicated below.

**Table:** 

		For mice	For human
TNF	Forward	CCCTCCAGAAAAGACACCATG	TCTCTCCACAGGCTTTAAGA
	Reverse	GCCACAAGCAGGAATGAGAAG	GGGCTTTGTTTATGTAGGGT
IL-1β	Forward	CCACCTTTTGACAGTGATGA	TCAGCCAATCTTCATTGCTC
	Reverse	GAGATTTGAAGCTGGATGCT	TTCATCTGTTTAGGGCCATC
IL-6	Forward	GTCGGAGGCTTAATTACACA	GAGTCTCAACCCCCAATAAA
	Reverse	TTTTCTGCAAGTGCATCATC	GAGAAGGAGTTCATAGCTGG
GPR110	Forward	CCAAGAGAAGCCAAACCTCC	GGACATATCAATACAAAGAAATGG
	Reverse	TTCGATAAGCCAGCAGGATG	AAAGAGCCGTCTTCTAATGG
GAPDH	Forward	CCACTCACGGCAAATTCAAC	TTCTATAAATTGAGCCCGCA
	Reverse	CTCCACGACATACTCAGCAC	AATACGACCAAATCCGTTGA
iNOS	Forward	CCTAGTCAACTGCAAGAGAA	
	Reverse	TTTCAGGTCACTTTGGTAGG	
CCL2	Forward	GGATCGGAACCAAATGAGAT	
	Reverse	ATTTACGGGTCAACTTCACA	
Ly6G	Forward	GGGCTGAGAGAAAGTAAAGT	
	Reverse	ACTTTGCAATGTGACAAGTG	
F4/80	Forward	TCCAAGATGGGTTAACATCC	
	Reverse	CAAAACTGCCATCAACTCAT	

### ROS assays

ROS were detected with the cell-permeable dye 2′,7′-dichlorofluoresceindiacetate (H2DCFDA) that is oxidized by hydrogen peroxide, peroxynitrite (ONOO-), and hydroxy radical (OH) to yield the fluorescent molecule 2′7′-dichlorofluorescein. Cells were plated and treated with LPS and synaptamide. After the culture medium was removed, the cells were washed with PBS and then incubated with 10 μM H2DCFDA in serum-free DMEM for 15 min at 37 °C and 5% CO_2_. After the cells were washed twice with PBS, the intracellular 2′7′-dichlorofluorescein was analyzed by fluorimetry at Excitation 488 nm/Emission 535 nm.

### Transmigration assay

Neutrophil migration was measured by the Boyden chamber (Cell Biolabs, San Diego, CA). The cells were resuspended in serum-free DMEM containing synaptamide (10 nM) and added to the upper compartment of the chamber. The lower compartment was filled with 30 nM LTB4 and 10 ng/mL CCL2 in 150 μL DMEM. The two compartments were separated by 3 μm polycarbonate membrane. After 30-min incubation with LTB4/CCL2 and synaptamide, cell migration to the lower chamber was detected using the CytoSelectTM migration assay kit (Cell Biolabs, San Diego, CA) according to the manufacturer’s instructions.

### ELISA assays for cytokine production

Following stimulation of the cells with LPS and/or synaptamide, supernatants were collected and assayed using sandwich ELISA with their well-matched antibodies. TNF, IL-1β, IL-6, and CCL2 were detected using ELISA kits (Invitrogen Life Technologies, Frederick, MD, USA) according to the manufacturer’s instructions [20].

### Western blot analysis

Proteins from cell lysates (50 μg protein) were separated via SDS–PAGE and electroblotted onto a polyvinylidene difluoride (PVDF) membrane for 90 min at 100 V at 4 °C. After blocking with 5% bovine serum albumin (BSA) for 60 min in TBS-T (20 mM Tris–HCl, pH 7.5, 50 mM NaCl, 0.1% Tween 20), the membrane was incubated overnight with primary antibodies in 5% BSA at 4 °C, washed with TBS-T buffer, and incubated for 60 min in anti-rabbit or anti-mouse IgG–horseradish peroxidase (Santa Cruz Biotechnology, Santa Cruz, CA, USA) secondary antibodies diluted in 5% BSA (1:3000). After treating with chemiluminescence reagents (Thermo Fisher Scientific, Rockford, IL, USA), protein bands were detected and quantified using a Kodak Gel Logic 440 imaging system with Image J software. Unless specified otherwise, glyceraldehyde 3-phosphate dehydrogenase (GAPDH) was used as the loading control for all western blotting analyses.

### Statistical analysis

The results are expressed as means ± SEM for triplicate determinations and represent at least three independent experiments. The statistical analysis included Student’s *t* test and a one-way ANOVA followed by Dunnett’s test for multiple comparisons. The mean differences were considered significant when *P* < 0.05.

## Results

### In vivo anti-neuroinflammatory effect of synaptamide is GPR110-dependent

**Fig. 1:**
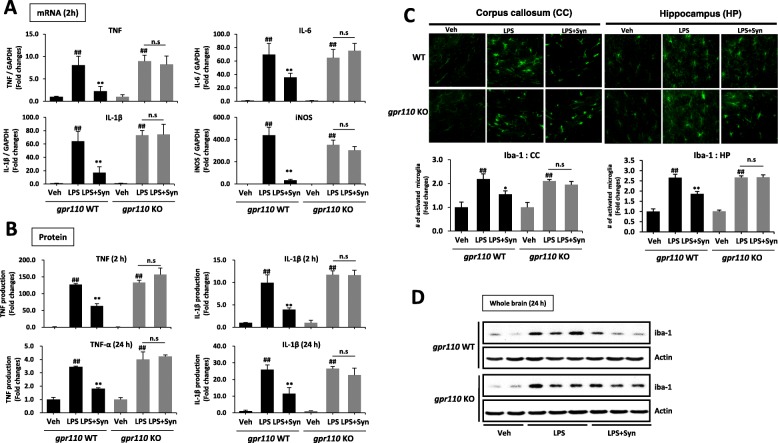
Anti-inflammatory effects of synaptamide in vivo require GPR110 receptor. Wild-type (WT) and GPR110 knock-out (KO) C57BL/6 mice (*n* = 3 for each group) were treated with synaptamide (5 mg/kg, i.p.) following LPS injection (1 mg/kg, i.p.), and brain and blood samples were collected to determine LPS-induced gene or protein expression at 2 and/or 24 h. The cytokine/chemokine mRNA expression in the brain was determined by qPCR at 2 h after LPS/synaptamide injection (**a**). The cytokine protein levels in blood were determined by ELISA at 2 and 24 h after LPS/synaptamide injection (**b**). Brain sections (30-μm-thick) were prepared 24 h after LPS/synaptamide injection and immunostained for Iba-1, and fluorescence microscopic images of the corpus callosum (CC) and hippocampus (HP) are presented along with quantification results of Iba-1 (**c**). The Iba-1 protein levels are shown by Western blot analysis of the whole brain extract (**d**). The cytokine protein levels in the blood were determined by ELISA at 2 and 24 h after LPS/synaptamide injection (**c**). Values are presented as mean ± SEM (*n* = 3) representing two independent experiments. ns, the difference of means is not statistically significant

We have previously reported that synaptamide suppresses LPS-induced neuroinflammation [18]. To test the involvement of GPR110 in synaptamide-mediated anti-neuroinflammatory responses, pro-inflammatory cytokine expression was compared between WT and GPR110 KO mice after the mice were injected with LPS (1 mg/kg, i.p.) followed by synaptamide (5 mg/kg, i.p.). At 2 h after LPS injection, mRNA levels of the proinflammatory genes TNF, IL-1β, IL-6, and iNOS in WT brain tissue increased 8.6 ± 2.9, 64.2 ± 14.7, 69.6 ± 16.5, and 439.6 ± 71.0 folds, respectively. Subsequent injection of synaptamide significantly dampened the LPS-induced mRNA expression of these mediators by 71.8, 73.3, 48.9, and 92.2%, respectively, in WT but not in GPR110 KO mice (Fig. 1a). At 2 and 24 h after LPS injection, the plasma protein level of TNF and IL-1β increased significantly by 127.2 ± 2.8 (2 h)/3.5 ± 0.1 (24 h) and 9.9 ± 1.8 (2 h)/25.9 ± 2.9 (24 h) fold, respectively (Fig. 1b). Like the case with the brain mRNA levels (Fig. 1a), synaptamide significantly suppressed the LPS-induced increase in plasma proinflammatory proteins at both time points in WT, but not in GPR110 KO mice (Fig. 1b). As an inflammatory marker, we compared the effect of synaptamide on the number of activated microglia in WT and GPR110 KO mice. Brain sections were prepared at 24 h after LPS and synaptamide injections and immunostained with antibody against the microglial marker Iba-1. In the WT and GPR110 KO mice, systemic LPS administration significantly increased the number of activated microglia in the corpus callosum and hippocampus (Fig. 1c). Other brain regions such as the cortex and thalamus also showed the similar trend of increase in microglia activation after LPS stimulation (Additional file 1: Figure S2). Subsequent synaptamide treatment significantly reduced LPS-induced iba-1 immunoactivity in WT mice, but synaptamide showed no effects in GPR110 KO mice. Western blot analysis also indicated that synaptamide significantly lowered the LPS-induced Iba-1 protein expression in the brains of WT, but not in GPR110 KO mice (Fig. 1d). These data indicated that the anti-neuroinflammatory effect of synaptamide in vivo is GPR110-dependent.

### GPR110 mediates the inhibitory effects of synaptamide on LPS-induced inflammatory responses in microglia

**Fig. 2:**
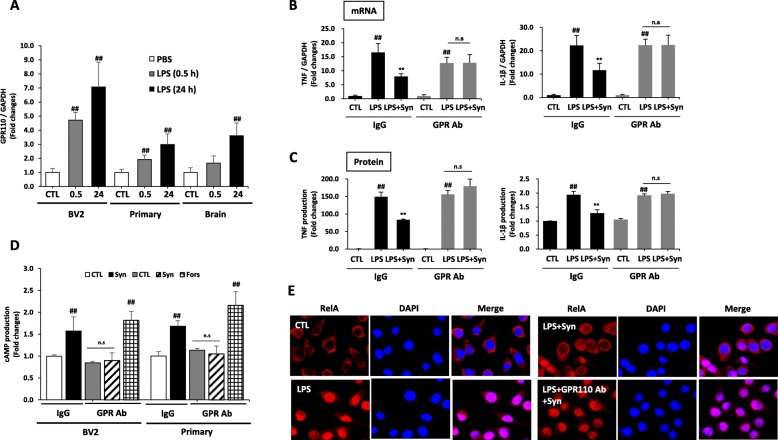
Blocking GPR110 activation abolishes the anti-inhibitory effects of synaptamide in microglia. Cells were incubated with LPS (100 ng/mL) for the indicated time, and GPR110 mRNA levels were determined by qPCR, showing LPS-induced elevation of GPR110 in BV2 murine microglia cell line, 8-week-old mice-derived primary adult microglia and 8-week-old mice whole brain (**a**). Control IgG and GPR110 *N*-terminal blocking antibody (300 ng/mL) were added 30 min prior to LPS treatment (100 ng/mL). Synaptamide (10 nM) was added immediately after LPS exposure. The mRNA levels of TNF and IL-1β were determined by qPCR after incubation for 1 h (**b**). After 24 h, accumulated levels of TNF and IL-1β in the media were measured by ELISA (**c**). Cells were treated with 10 nM synaptamide or 10 μM forskolin (Fors) as a positive control for 15 min, and cAMP levels were measured. Translocation of RelA (p65) was determined in BV2 cells by immunocytochemistry (red, RelA; blue, DAPI) (**e**). The data are expressed as the mean ± SEM (*n* = 3) representing at least three independent experiments. ns, the difference of means is not statistically significant

Although the GPR110 expression in the brain is high during development, its level diminishes afterwards [16], raising a question about the significance of GPR110 in the inhibitory effects of synaptamide on neuroinflammation in the adult stage. Therefore, we first examined the possibility of GPR110 induction in response to LPS in microglia cells that are known to be the major player for inflammation in the brain. We found that GPR110 expression was indeed increased in microglia cells and brain tissue in as little as 30 min after LPS treatment (Fig. 2a), suggesting that the GPR110 receptor can be upregulated by inflammation for host defense in the brain. The role of GPR110 in the synaptamide-induced anti-inflammatory responses was tested in vitro using the *N*-terminal targeting GPR110 antibody that was shown to block ligand binding [16]. Microglial cells were pre-treated with 300 ng/mL GPR110 antibody or IgG control for 30 min prior to LPS and synaptamide treatment. Synaptamide significantly suppressed LPS-induced TNF and IL-1β mRNA expression in the IgG control; however, the GPR110 antibody completely blocked the inhibitory effect of synaptamide on TNF and IL-1β expression at 2 h after LPS treatment (Fig. 2b). At 24 h after treatment, LPS-induced increases in TNF and IL-1β protein were also significantly suppressed by synaptamide in culture medium, and the suppression was blocked by the GPR110 antibody (Fig. 2c). Previous studies showed that cAMP/PKA signaling attenuates LPS-induced inflammatory responses by inhibiting NF-κB activation [21, 22], and that synaptamide-mediated inhibition of the LPS-induced RelA nuclear translocation and proinflammatory responses also were cAMP/PKA-dependent [20]. Therefore, we further tested whether GPR110 activation mediates synaptamide-induced cAMP production and inhibition of NF-κB activation in microglia cells. The *N*-terminal targeting GPR110 antibody completely blocked synaptamide-induced cAMP elevation (Fig. 2d) and inhibition of RelA (p65) translocation to nucleus (Fig. 2e). These results suggest that GPR110 activation is essential for synaptamide to block LPS-induced NF-kB activation and thus suppress pro-inflammatory responses in microglia.

### Synaptamide suppresses LPS-induced inflammatory responses of peripheral innate immune cells in a GPR110/cAMP-dependent manner

**Fig. 3:**
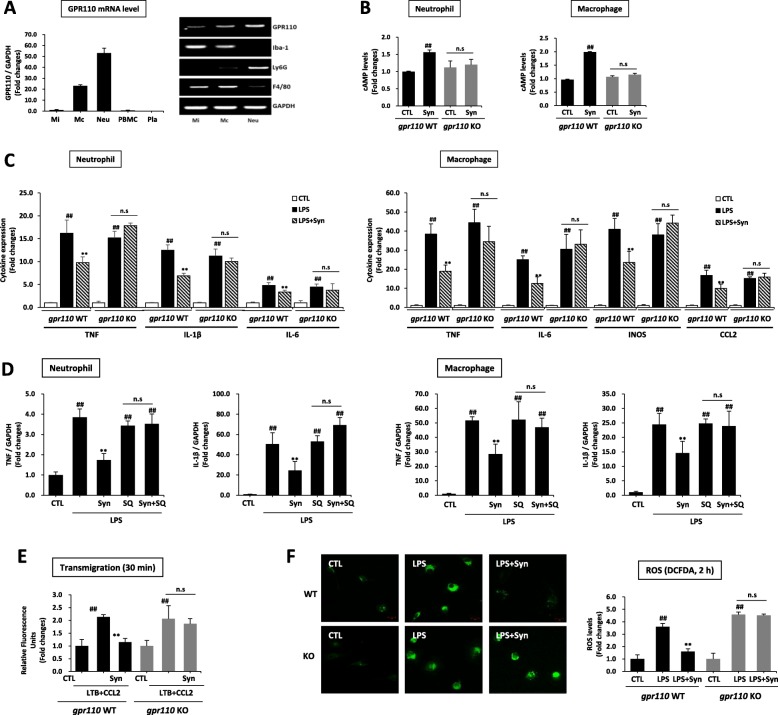
Synaptamide suppresses LPS-induced inflammatory responses in neutrophils and macrophages in a GPR110/cAMP-dependent manner. Levels of GPR110 mRNA were determined by qPCR in primary microglia (Mi), peritoneal macrophage (Mc), neutrophil (Neu), peripheral blood mononuclear cells (PBMC), and platelets (Pla). Cell purity was determined by PCR using specific marker proteins (Iba-1 for microglia; Ly6G for neutrophil, F4/80 for macrophage) (**a**). WT- and GPR110 KO-derived neutrophils and macrophages were treated with 10 nM synaptamide for 15 min, and cAMP levels were measured (**b**). The cytokine/chemokine expression in the neutrophils and macrophages was determined by qPCR at 1 h after treatment of 100 ng/mL LPS followed by 10 nM synaptamide (**c**). Adenylyl cyclase inhibitor (SQ 22536, 10 μM) was added 30 min prior to LPS/synaptamide treatment. Levels of TNF and IL-1β mRNA were determined by qPCR after 1-h incubation in neutrophils and macrophages (**d**). Activity of transmigration was determined by Boyden chamber assay (at 30 min) after incubation. WT and GPR110 KO-derived neutrophils were incubated with LTB4 (30 nM)/CCL2 (10 ng/mL) or LPS (100 ng/mL) and/or 10 nM synaptamide (**e**). WT and GPR110 KO-derived peritoneal macrophages were incubated with LPS (100 ng/mL) and synaptamide (10 nM) for 2 h, and intracellular ROS level was determined by immunocytochemistry and ELISA using H2DCFDA (10 μM) (**f**). The data are expressed as the mean ± SEM (*n* = 3) representing at least three independent experiments. ns, the difference of means is not statistically significant

Intraperitoneally injected synaptamide has been detected in the plasma as well as in the brain [18], strongly suggesting that synaptamide may also act on peripheral immune cells for suppressing immune responses. However, the role of GPR110 in innate immune cells during acute inflammation is unclear. To find possible target cells for synaptamide action, we first examined the GPR110 expression on several mouse innate immune cells. In contrast to peripheral blood mononuclear cells (PBMC) and platelets, neutrophils and macrophages highly expressed GPR1110 (Fig. 3a). To further determine whether these cells respond to synaptamide, we measured the synaptamide-induced cAMP increase, a downstream second messenger of GPR110 activation. Synaptamide significantly elevated the intracellular cAMP level in WT mouse neutrophils and peritoneal macrophages (Fig. 3b). However, synaptamide did not increase cAMP in GPR110 KO mouse-derived neutrophils or macrophages (Fig. 3b). The LPS-induced proinflammatory cytokine/chemokine expression was significantly suppressed by synaptamide in WT- but not in GPR110 KO-derived neutrophils and macrophages (Fig. 3c). Similarly, human neutrophils highly expressed GPR110 (Additional file 1: Figure S3A) while PBMC and platelets did not, and incubation of the neutrophils with synaptamide increased cAMP (Additional file 1: Figure S3B) and suppressed LPS-induced proinflammatory cytokines TNF, IL-1b, and IL-6 (Additional file 1: Figure S3C). In addition, the anti-inflammatory effect of synaptamide was abolished by an adenylyl cyclase inhibitor, SQ 22536, as shown in Fig. 3d for the LPS-induced TNF and IL-1β mRNA expression in both neutrophils and macrophages. LPS also elicited innate immune responses such as transmigration of neutrophils (Fig. 3e) and ROS production in macrophages (Fig. 3f). Synaptamide in a GPR110-dependent manner significantly inhibited neutrophil migration (Fig. 3e) and ROS production in macrophages (Fig. 3f) evaluated at 30 min and 2 h after LPS treatment, respectively. These results indicate that synaptamide suppresses LPS-induced proinflammatory responses also in peripheral cells including neutrophils and macrophages by activating GPR110/cAMP signaling.

### Synaptamide suppresses TNF-challenged proinflammatory responses

**Fig. 4:**
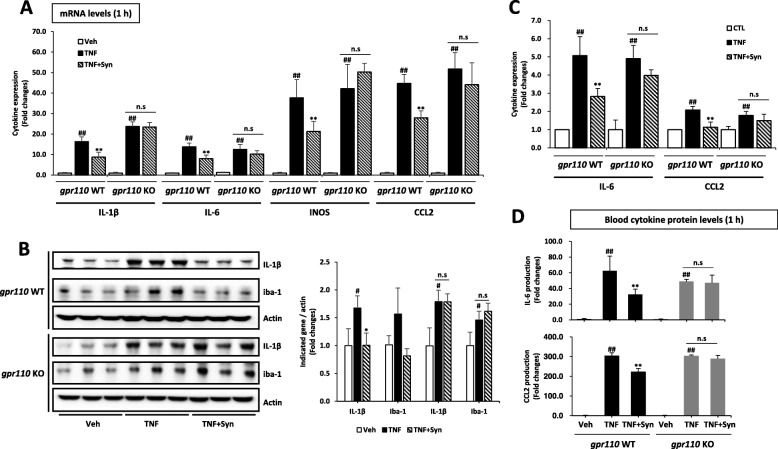
Synaptamide suppresses TNF-challenged pro-inflammatory responses. Mice were injected with synaptamide (5 mg/kg, i.p.) following TNF injection (250 μg /kg, i.v.), and brain and blood samples were collected after 1 h to determine inflammatory gene expression by qPCR (**a**) and the protein level in the whole brain by the Western blot analysis with quantitative results (**b**). Primary adult microglia cells were stimulated with TNF (20 ng/mL) followed by 10 nM synaptamide for 1 h (**c**). The protein level in the blood was analyzed by ELISA (**d**). Values are presented as mean ± SEM (*n* = 3). ns, the difference of means is not statistically significant

The systemic administration of LPS produces an acute inflammatory response in the brain as indicated in Fig. 1 even though LPS cannot cross the BBB. It has been reported that cytokines such as TNF and IL-6 contained in the circulation can reach the brain by saturable transport systems or transporter/receptors [9, 23]. Therefore, the modulation of neuroinflammatory responses may occur as a secondary effect of the proinflammatory cytokines in the plasma, which are significantly elevated after LPS injection and inhibited by synaptamide via a GPR110-dependent mechanism (Fig. 1c). We tested this possibility under a TNF-challenged condition. As expected, intravenous injection of TNF (250 μg/kg) increased the mRNA expression of proinflammatory mediators IL-1β, IL-6, iNOS, and CCL2 in the brain by 16.3 ± 2.3, 13.8 ± 1.9, 37.7 ± 8.8, and 44.7 ± 4.4 folds, respectively. The subsequent injection of synaptamide (5 mg/kg, i.p.) significantly lowered the expression of IL-1β, IL-6, iNOS, and CCL2 in the brain by 46.4, 42.4, 43.7, and 37.6%, respectively, in WT but not in GPR110 KO mice (Fig. 4a). The western blot with quantitative analysis also showed that TNF markedly induced proinflammatory marker proteins such as IL-1β and Iba-1 in the brain tissue and that these proteins were significantly suppressed in a GPR110-dependent manner when the mice were treated with synaptamide (Fig. 4b). Similar results were observed in isolated microglial cells obtained from adult WT and GPR110 KO mice. At 1 h after treatment with TNF (20 ng/mL), the mRNA expression of proinflammatory mediators IL-6 and CCL2 was significantly increased in both WT- and KO-derived microglia, but this increase was attenuated by synaptamide (10 nM) only in WT-derived microglia (Fig. 4c). In the plasma, the IL-6 and CCL2 protein levels were also elevated after TNF challenge, and this increase was partially blocked by the subsequent injection of synaptamide in a GPR110-dependent manner (Fig. 4d). These data indicated a direct link between circulating inflammatory mediators and neuroinflammatory responses, suggesting that synaptamide-induced GPR110 activation in both brain microglia and peripheral immune cells plays a significant role in suppressing neuroinflammation.

## Discussion

In this study, we demonstrate that LPS rapidly induces GPR110 in the adult brain and that the activation of GPR110/cAMP signaling is essential for the anti-inflammatory effects of synaptamide. Under the LPS-induced systemic inflammatory condition, synaptamide inhibited neuroinflammation by activating GPR110 in peripheral and brain immune cells where GPR110 expression is either high or elevated after LPS challenge, respectively.

Neuroinflammatory mechanisms are increasingly implicated in the pathogenesis of neurodegenerative diseases such as Alzheimer’s disease, Parkinson’s disease, and traumatic brain injury [24–26]. Microglia, as potent immune effector cells in the brain, produce and release proinflammatory and cytotoxic mediators such as cytokines, chemokines, and reactive oxygen species upon activation, leading to compromised neuronal survival and function [27–29]. We demonstrated in this study that activation of GPR110 (ADGRF1), the target receptor of synaptamide, suppressed inflammatory responses caused by LPS through a cAMP-dependent process. The immune regulatory nature of GPR110 activation observed in not only microglia but also peripheral innate immune cells collectively contributed to the anti-neuroinflammatory effects of synaptamide under a systemic inflammatory condition.

G protein-coupled receptors (GPCRs) are major therapeutic targets for a variety of diseases including inflammation as they regulate a wide range of physiologic and pathophysiologic processes. In recent years, growing evidence indicated the involvement of aGPCR in inflammation [12]. For example, EMR2 (ADGRE2) was shown to regulate neutrophil adhesion, ROS production, and migration [30] while the activation of GPR97 (ADGRG3) suppresses inflammation via crosstalk between Gα_s_ and Gα_i_ in peripheral mononuclear cells [31]. Our current study revealed that GPR110 is a new aGPCR member transmitting anti-inflammatory signals by elevating cAMP which is a well-established regulator of innate and adaptive immune responses [32]. Synaptamide, an endogenous ligand of GPR110 [17], suppressed LPS-induced pro-inflammatory cytokine expression both in vivo (Figs. 1 and 4) and in vitro (Figs. 2 and 3) only in the presence of GPR110, indicating the necessity of GPR110 activation for the anti-inflammatory action of synaptamide. It is well established that the inflammatory response is coordinated by hundreds of genes that promote host defense against injury or infection [33–35]. The rapid induction of GPR110 upon LPS challenge in microglia and brain (Fig. 2), where GPR110 expression is diminished in the adult stage [17], suggests that GPR110 induction is a part of the neuroprotective mechanism, stimulating immune regulatory defense capacity in the brain.

Even though brain has long been considered immunologically protected by the BBB [36, 37], recent studies have shown that the central nervous and the peripheral immune systems can communicate via diverse means such as alteration of the BBB [38, 39], release of microbial short-chain fatty acids [40, 41], or exposure to proinflammatory cytokines from the circulatory system [42] [23]. LPS cannot cross the BBB, but numerous studies in animals demonstrated that acute systemic exposure to LPS induces pro-inflammatory cytokine expression in the brain [43, 44]. Under this condition, peripheral immune cells including macrophages and neutrophils are known to play major roles in the onset and maintenance of systemic and brain inflammation [45]. The high GPR110 expression, GPR110-dependent cAMP increase, and inhibition of LPS-induced cytokine expression in neutrophils and macrophages (Fig. 3a-c) suggest that these cells are among the peripheral targets of synaptamide that contribute to the anti-neuroinflammatory effects of synaptamide. Other peripheral immune targets that produce and release inflammatory mediators such as epithelial [46, 47] or innate lymphoid cells [48, 49] may also play a role in GPR110-dependent anti-neuroinflammatory effects in the LPS-induced systemic inflammatory condition, and this possibility requires further investigation.

Neutrophil migration [50] and ROS production by macrophages are critical components of the host defense mechanism [51]. Nevertheless, unregulated activation of neutrophils or macrophages can be detrimental to the host and cause tissue injury. Systemic LPS models are often used to replicate uncontrolled inflammation associated with sepsis and neurodegenerative or autoimmune diseases [52–55]. The GPR110-dependent inhibition of neutrophil migration and macrophage ROS production observed in the LPS model (Fig. 3e, f) suggests that GPR110 activation may protect against tissue or organ damage caused by overactive neutrophils or macrophages under uncontrolled inflammatory conditions.

**Fig. 5:**
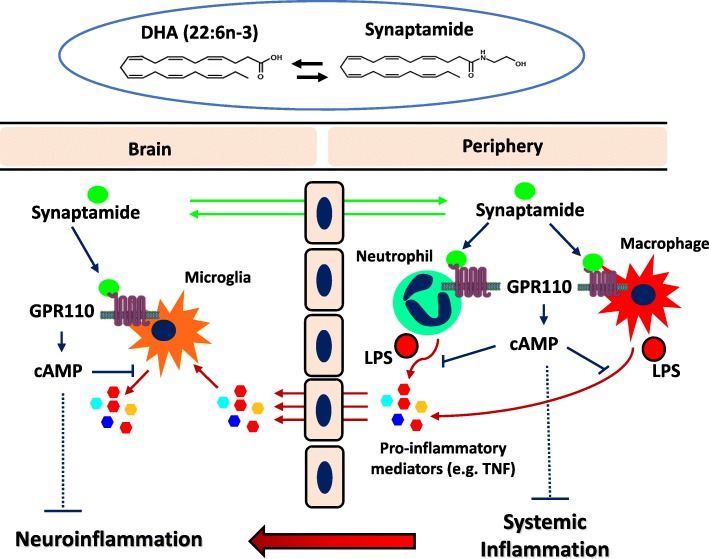
Proposed model for synaptamide/GPR110-mediated suppression of LPS-induced neuroinflammation. LPS induces activation of peripheral immune cells such as neutrophils and macrophages, which increases the level of pro-inflammatory mediators. Under systemic inflammatory conditions, inflammatory molecules produced by peripheral immune cells traverse the blood-brain barrier and activate microglia causing neuroinflammation. Pharmacologically administered synaptamide acts on central and peripheral targets where GPR110 is expressed or induced after LPS challenge to ameliorate neuroinflammation through cAMP-dependent immune regulatory mechanisms. Synaptamide endogenously produced from DHA in the brain or peripheral tissues can also be protective

Once activated, the peripheral innate immune cells secrete a variety of mediators like cytokines, reactive oxygen species, or chemokines that regulate the microenvironment and determine the direction of the immune responses [56–58]. The circulating cytokines in turn cause neuroinflammation after transport to the brain [10, 59, 60], which is consistent with the pro-inflammatory responses induced by TNF in cultured microglial cells and in the brain after intravenous injection in our current study (Fig. 4). In such case, LPS-induced neuroinflammation is a secondary effect of peripheral pro-inflammatory cytokine production [2], and both peripheral and central immune systems that can transmit the GPR110/cAMP signal are targets for the anti-inflammatory effects of synaptamide. Figure 5 presents a proposed model for GPR110-mediated suppression of LPS-induced neuroinflammation. Through activating Gα_s_-coupled GPR110, intraperitoneally injected synaptamide first elevates the cAMP level and suppresses peripheral pro-inflammatory cytokine production by immune cells that include neutrophils and macrophages. Subsequently, the circulating pro-inflammatory cytokines enter the brain and activate microglia [10, 59, 60]. Synaptamide also enters the brain [18] and inhibits microglial production of additional pro-inflammatory mediators through GPR110/cAMP signaling. The GPR110-dependent pharmacological action of synaptamide in both central and peripheral immune cells cooperatively ameliorates neuroinflammation caused by systemic LPS administration. This proposed mechanism may explain the generally better anti-neuroinflammatory effects of synaptamide observed in vivo (Fig. 1a) compared to in vitro effects on individual cells (Figs. 2b, 3c). By the same mechanism, synaptamide produced endogenously from DHA under challenged conditions may also have a neuroprotective role by acting on these innate immune cells.

## Conclusions

We have demonstrated that activation of GPR110 by synaptamide exerts an anti-neuroinflammatory function by upregulating cAMP-dependent signaling in microglia and innate peripheral immune cells under LPS- or TNF-α-stimulated conditions. The necessity of GPR110 for the anti-inflammatory effects of synaptamide revealed GPR110 as a new GPCR target for immune regulatory function. We propose that GPR110-mediated inhibition of innate immune cell activation may serve as a new therapeutic strategy for controlling brain and/or peripheral inflammation and related diseases.

## Footnote

The term “synaptamide” instead of “DHEA” was used for *N*-docosahexaenoylethanolamine since DHEA is a widely used and accepted term for the steroid, dehydroepiandrosterone.

## Supplementary information

**Additional file 1: Figure S1.** FACS analysis showing no macrophage contamination in microglia preparation. Microglia and peritoneal macrophages isolated from 8 weeks old normal mice were labeled with CD11 and CD45 and analyzed by flow cytometry. The CD11b ^+^/CD45 ^low^ microglia cell population shows no overlap with CD11b ^+^ / CD45 ^high^ macrophage population, indicating that the microglia cell preparation was not contaminated with macrophages. **Figure S2.** Fluorescence microscopic images of the Cortex (CX), hippocampus (HP) and Thalamus (TH) obtained from brain sections prepared 24 h after LPS/synaptamide injection and immunostained for Iba-1. LPS increased Iba-1 staining while synaptamide injection prevented the LPS effect. Scale bar: 500 μm. **Figure S3.** Synaptamide increases cAMP production and suppresses LPS-induced inflammatory responses in human neutrophils where GPR110 is highly expressed. Levels of GPR110 mRNA were determined by qPCR in neutrophils (Neu), peripheral blood mononuclear cells (PBMC), and platelets (Pla) isolated from healthy donors (A). Neutrophils were treated with 10 nM synaptamide and 10 μM forskolin (For) for 15 min, and the cAMP level was measured (B). The cytokine expression in the neutrophils was determined by qPCR at 1 h after treatment of 100 ng/mL LPS followed by 10 nM synaptamide (C).

## Data Availability

The data supporting the conclusions of this study will be available upon request.

## Abbreviations

ANOVA: Analysis of variance
BBB: Blood-brain barrier
cAMP: Cyclic adenosine monophosphate
CCL2: Chemokine (C-C motif) ligand 2
CNS: Central nervous system
DAPI: 4′,6-Diamidino-2-phenylindole
DHA: Docosahexaenoic acid
ELISA: Enzyme-linked immunosorbent assay
GAPDH: Glyceraldehyde-3-phosphate
GPCR: G-protein-coupled receptor
GPR110: G-protein-coupled receptor 110
Iba-1: Ionized calcium-binding adaptor molecule 1
IL: Interleukin
iNOS: Inducible nitric oxide synthase
KO: Knock out
LPS: Lipopolysaccharide
NF-κB: Nuclear factor kappa light chain enhancer of activated B cells
PKA: Protein kinase A
qPCR: Quantitative polymerase chain reaction
SEM: Standard error of mean
TNF: Tumor necrosis factor

## References

[1] Chen WW, Zhang X, Huang WJ (2016). Role of neuroinflammation in neurodegenerative diseases (review). Mol Med Rep.

[2] Qin L (2007). Systemic LPS causes chronic neuroinflammation and progressive neurodegeneration. Glia.

[3] Tanaka S (2013). Activation of microglia induces symptoms of Parkinson’s disease in wild-type, but not in IL-1 knockout mice. J Neuroinflammation.

[4] Yan J (2014). Inflammatory response in Parkinson’s disease (review). Mol Med Rep.

[5] Dantzer R (2004). Cytokine-induced sickness behaviour: a neuroimmune response to activation of innate immunity. Eur J Pharmacol.

[6] Gatti S, Bartfai T (1993). Induction of tumor necrosis factor-alpha mRNA in the brain after peripheral endotoxin treatment: comparison with interleukin-1 family and interleukin-6. Brain Res.

[7] Banks WA, Kastin AJ, Durham DA (1989). Bidirectional transport of interleukin-1 alpha across the blood-brain barrier. Brain Res Bull.

[8] De Laere M (2017). Increased transendothelial transport of CCL3 is insufficient to drive immune cell transmigration through the blood-brain barrier under inflammatory conditions in vitro. Mediat Inflamm.

[9] Cazareth J (2014). Molecular and cellular neuroinflammatory status of mouse brain after systemic lipopolysaccharide challenge: importance of CCR2/CCL2 signaling. J Neuroinflammation.

[10] Pan W, Kastin AJ (2002). TNFalpha transport across the blood-brain barrier is abolished in receptor knockout mice. Exp Neurol.

[11] Pan W (2007). TNFalpha trafficking in cerebral vascular endothelial cells. J Neuroimmunol.

[12] Lin HH (2017). Adhesion GPCRs in regulating immune responses and inflammation. Adv Immunol.

[13] Ho MK (2009). Regulation of transcription factors by heterotrimeric G proteins. Curr Mol Pharmacol.

[14] Shi G (2007). Identification of an alternative G {alpha}q-dependent chemokine receptor signal transduction pathway in dendritic cells and granulocytes. J Exp Med.

[15] Sun L, Ye RD (2012). Role of G protein-coupled receptors in inflammation. Acta Pharmacol Sin.

[16] Lee JW (2016). Orphan GPR110 (ADGRF1) targeted by N-docosahexaenoylethanolamine in development of neurons and cognitive function. Nat Commun.

[17] Kim HY, Spector AA (2018). N-Docosahexaenoylethanolamine: a neurotrophic and neuroprotective metabolite of docosahexaenoic acid. Mol Asp Med.

[18] Park T (2016). N-Docosahexaenoylethanolamine ameliorates LPS-induced neuroinflammation via cAMP/PKA-dependent signaling. J Neuroinflammation.

[19] Livak KJ, Schmittgen TD (2001). Analysis of relative gene expression data using real-time quantitative PCR and the 2(-Delta Delta C(T)) Method. Methods.

[20] Gisch N (2013). Structural reevaluation of Streptococcus pneumoniae Lipoteichoic acid and new insights into its immunostimulatory potency. J Biol Chem.

[21] Takahashi N (2002). Inhibition of the NF-kappaB transcriptional activity by protein kinase A. Eur J Biochem.

[22] Milne GR, Palmer TM (2011). Anti-inflammatory and immunosuppressive effects of the A2A adenosine receptor. ScientificWorldJournal.

[23] Banks WA (2005). Blood-brain barrier transport of cytokines: a mechanism for neuropathology. Curr Pharm Des.

[24] Loane DJ, Kumar A (2016). Microglia in the TBI brain: the good, the bad, and the dysregulated. Exp Neurol.

[25] Hansen DV, Hanson JE, Sheng M (2018). Microglia in Alzheimer’s disease. J Cell Biol.

[26] Martinez B, Peplow PV (2018). Neuroprotection by immunomodulatory agents in animal models of Parkinson’s disease. Neural Regen Res.

[27] Rose J (2017). Mitochondrial dysfunction in glial cells: implications for neuronal homeostasis and survival. Toxicology.

[28] Ising C, Heneka MT (2018). Functional and structural damage of neurons by innate immune mechanisms during neurodegeneration. Cell Death Dis.

[29] Fricker M (2018). Neuronal cell death. Physiol Rev.

[30] Yona S (2008). Ligation of the adhesion-GPCR EMR2 regulates human neutrophil function. FASEB J.

[31] Hsiao CC (2018). The adhesion G protein-coupled receptor GPR97/ADGRG3 is expressed in human granulocytes and triggers antimicrobial effector functions. Front Immunol.

[32] Raker VK, Becker C, Steinbrink K (2016). The cAMP pathway as therapeutic target in autoimmune and inflammatory diseases. Front Immunol.

[33] Sousa C, et al. Single-cell transcriptomics reveals distinct inflammation-induced microglia signatures. EMBO Rep. 2018;19(11). 10.15252/embr.201846171 PMC6216255 30206190

[34] Kim YS, Joh TH (2006). Microglia, major player in the brain inflammation: their roles in the pathogenesis of Parkinson's disease. Exp Mol Med.

[35] Crotti A, Ransohoff RM (2016). Microglial physiology and pathophysiology: insights from genome-wide transcriptional profiling. Immunity.

[36] Carson MJ (2006). CNS immune privilege: hiding in plain sight. Immunol Rev.

[37] Forrester JV, McMenamin PG, Dando SJ (2018). CNS infection and immune privilege. Nat Rev Neurosci.

[38] Banks WA, Erickson MA (2010). The blood-brain barrier and immune function and dysfunction. Neurobiol Dis.

[39] Banks WA (2015). Lipopolysaccharide-induced blood-brain barrier disruption: roles of cyclooxygenase, oxidative stress, neuroinflammation, and elements of the neurovascular unit. J Neuroinflammation.

[40] Bienenstock J, Kunze W, Forsythe P (2015). Microbiota and the gut-brain axis. Nutr Rev.

[41] Morris G (2017). The role of the microbial metabolites including tryptophan catabolites and short chain fatty acids in the pathophysiology of immune-inflammatory and neuroimmune disease. Mol Neurobiol.

[42] Banks WA (2006). The blood-brain barrier as a regulatory interface in the gut-brain axes. Physiol Behav.

[43] Laye S (1994). Peripheral administration of lipopolysaccharide induces the expression of cytokine transcripts in the brain and pituitary of mice. Brain Res Mol Brain Res.

[44] Brugg B (1995). Inflammatory processes induce beta-amyloid precursor protein changes in mouse brain. Proc Natl Acad Sci U S A.

[45] Rahat MA (2016). Macrophages and neutrophils: regulation of the inflammatory microenvironment in autoimmunity and cancer. Mediat Inflamm.

[46] Lotz M (2007). Cytokine-mediated control of lipopolysaccharide-induced activation of small intestinal epithelial cells. Immunology.

[47] Guillot L (2004). Response of human pulmonary epithelial cells to lipopolysaccharide involves Toll-like receptor 4 (TLR4)-dependent signaling pathways: evidence for an intracellular compartmentalization of TLR4. J Biol Chem.

[48] Duffin R (2016). Prostaglandin E (2) constrains systemic inflammation through an innate lymphoid cell-IL-22 axis. Science.

[49] Jacquelot N, Luong K, Seillet C (2019). Physiological regulation of innate lymphoid cells. Front Immunol.

[50] Tan SY, Weninger W (2017). Neutrophil migration in inflammation: intercellular signal relay and crosstalk. Curr Opin Immunol.

[51] Slauch JM (2011). How does the oxidative burst of macrophages kill bacteria? Still an open question. Mol Microbiol.

[52] Doi K (2009). Animal models of sepsis and sepsis-induced kidney injury. J Clin Invest.

[53] Liu M, Bing G (2011). Lipopolysaccharide animal models for Parkinson’s disease. Parkinsons Dis.

[54] Zakaria R (2017). Lipopolysaccharide-induced memory impairment in rats: a model of Alzheimer's disease. Physiol Res.

[55] Nogai A (2005). Lipopolysaccharide injection induces relapses of experimental autoimmune encephalomyelitis in nontransgenic mice via bystander activation of autoreactive CD4+ cells. J Immunol.

[56] Boettcher S, Manz MG (2017). Regulation of inflammation- and infection-driven hematopoiesis. Trends Immunol.

[57] Kumar V (2018). Dendritic cells in sepsis: potential immunoregulatory cells with therapeutic potential. Mol Immunol.

[58] Galdiero MR (2013). Tumor associated macrophages and neutrophils in tumor progression. J Cell Physiol.

[59] Varatharaj A, Galea I (2017). The blood-brain barrier in systemic inflammation. Brain Behav Immun.

[60] Erickson MA, Dohi K, Banks WA (2012). Neuroinflammation: a common pathway in CNS diseases as mediated at the blood-brain barrier. Neuroimmunomodulation.

