# Supplementary material for: GPR110 (ADGRF1) mediates anti-inflammatory effects of N-docosahexaenoylethanolamine
Source: J Neuroinflammation. 2019 Nov 15;16:225. doi: 10.1186/s12974-019-1621-2 (PMC6858791; doi:10.1186/s12974-019-1621-2)
Supplement: Supplementary file 1 — Additional file 1: Figure S1. FACS analysis showing no macrophage contamination in microglia preparation. Microglia and peritoneal macrophages isolated from 8 weeks old normal mice were labeled with CD11 and CD45 and analyzed by flow cytometry. The CD11b +/CD45 low microglia cell population shows no overlap with CD11b + / CD45 high macrophage population, indicating that the microglia cell preparation was not contaminated with macrophages. Figure S2. Fluorescence microscopic images of the Cortex (CX), hippocampus (HP) and Thalamus (TH) obtained from brain sections prepared 24 h after LPS/synaptamide injection and immunostained for Iba-1. LPS increased Iba-1 staining while synaptamide injection prevented the LPS effect. Scale bar: 500 μm. Figure S3. Synaptamide increases cAMP production and suppresses LPS-induced inflammatory responses in human neutrophils where GPR110 is highly expressed. Levels of GPR110 mRNA were determined by qPCR in neutrophils (Neu), peripheral blood mononuclear cells (PBMC), and platelets (Pla) isolated from healthy donors (A). Neutrophils were treated with 10 nM synaptamide and 10 μM forskolin (For) for 15 min, and the cAMP level was measured (B). The cytokine expression in the neutrophils was determined by qPCR at 1 h after treatment of 100 ng/mL LPS followed by 10 nM synaptamide (C). [file 12974_2019_1621_MOESM1_ESM.pptx]

## Slide 1
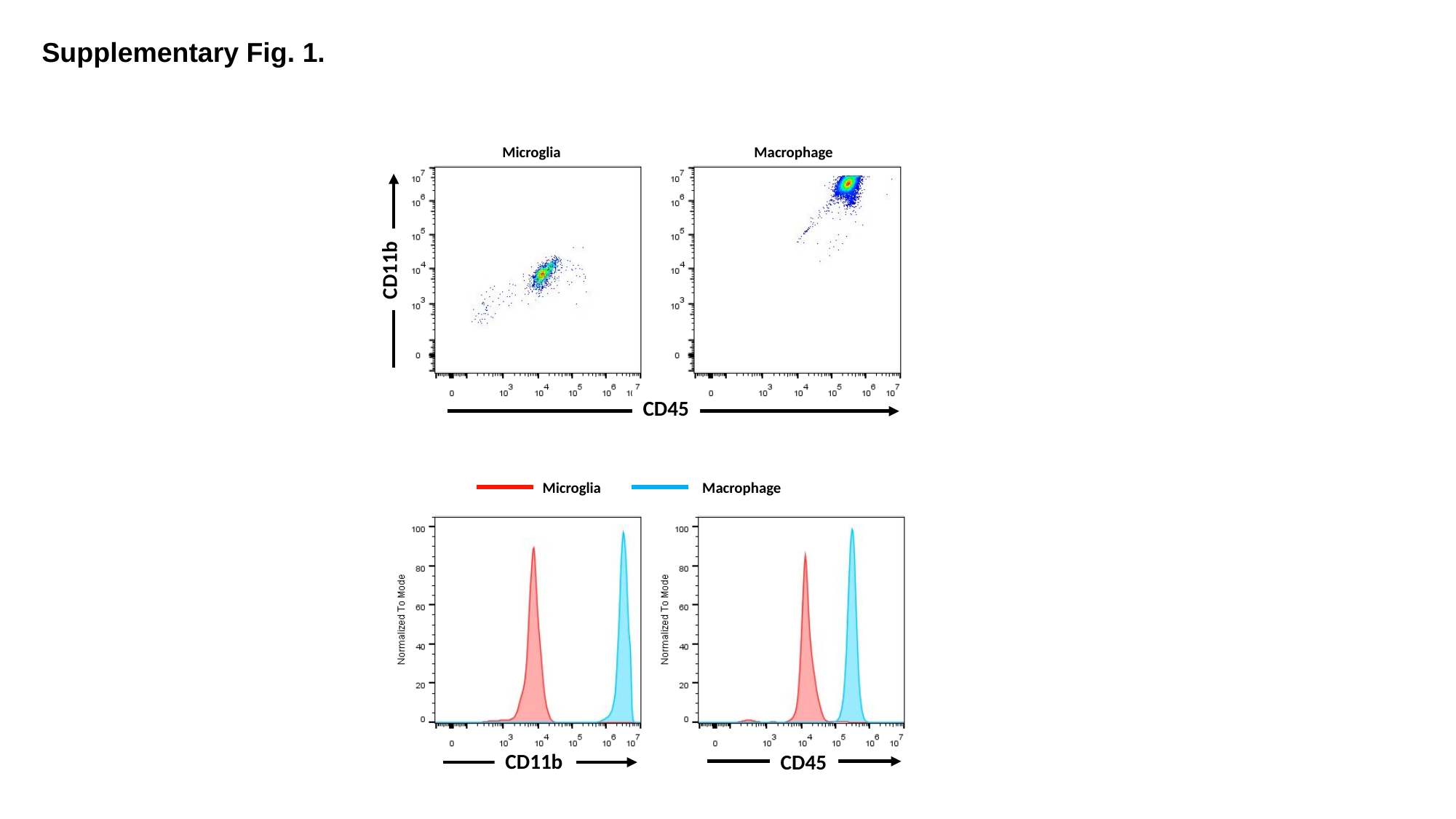

Supplementary Fig. 1.
Microglia
Macrophage
CD11b
CD45
Microglia
Macrophage
CD11b
CD45

## Slide 2
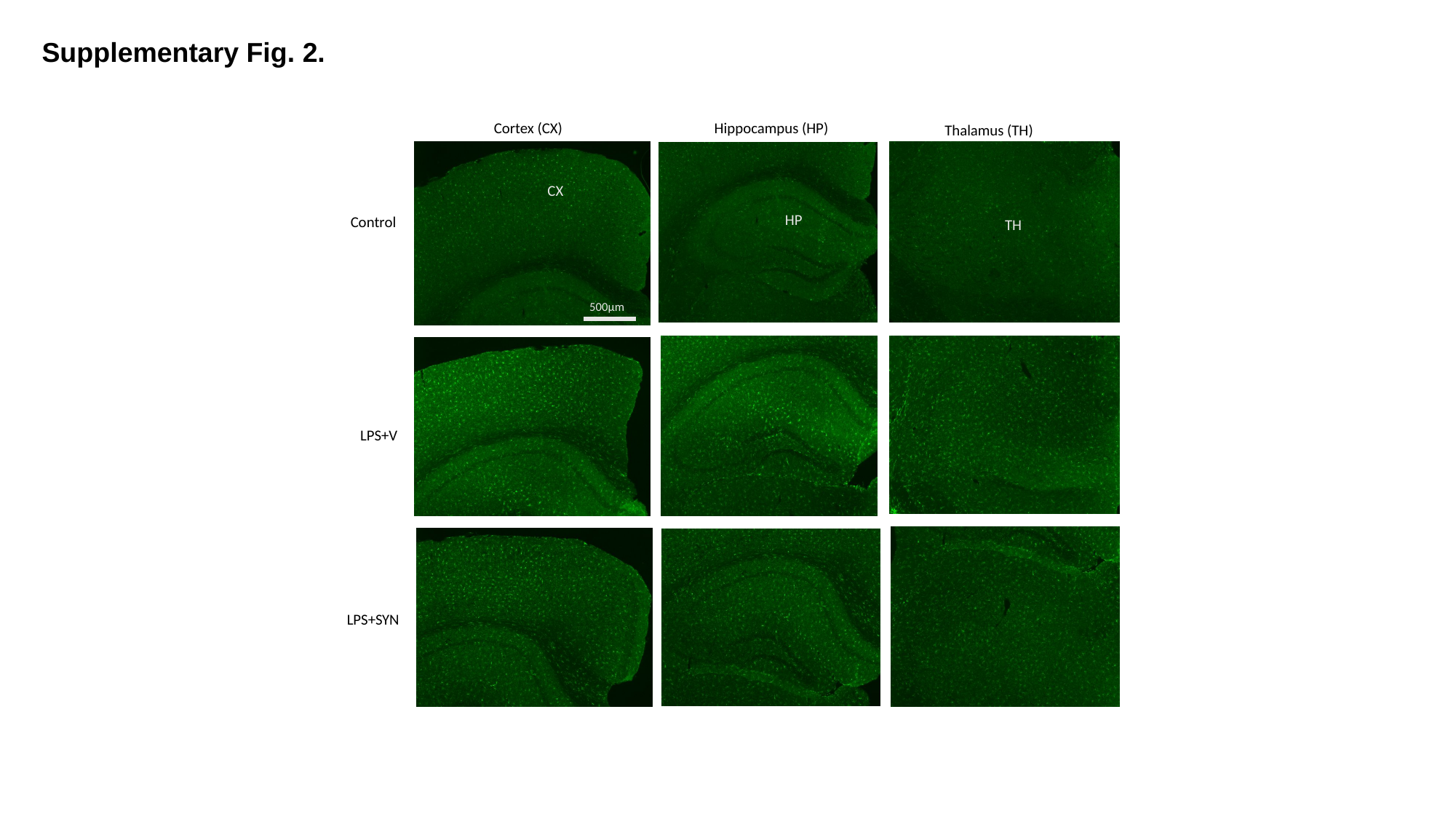

Supplementary Fig. 2.
Hippocampus (HP)
Cortex (CX)
Thalamus (TH)
CX
500µm
TH
HP
Control
LPS+V
LPS+SYN

## Slide 3
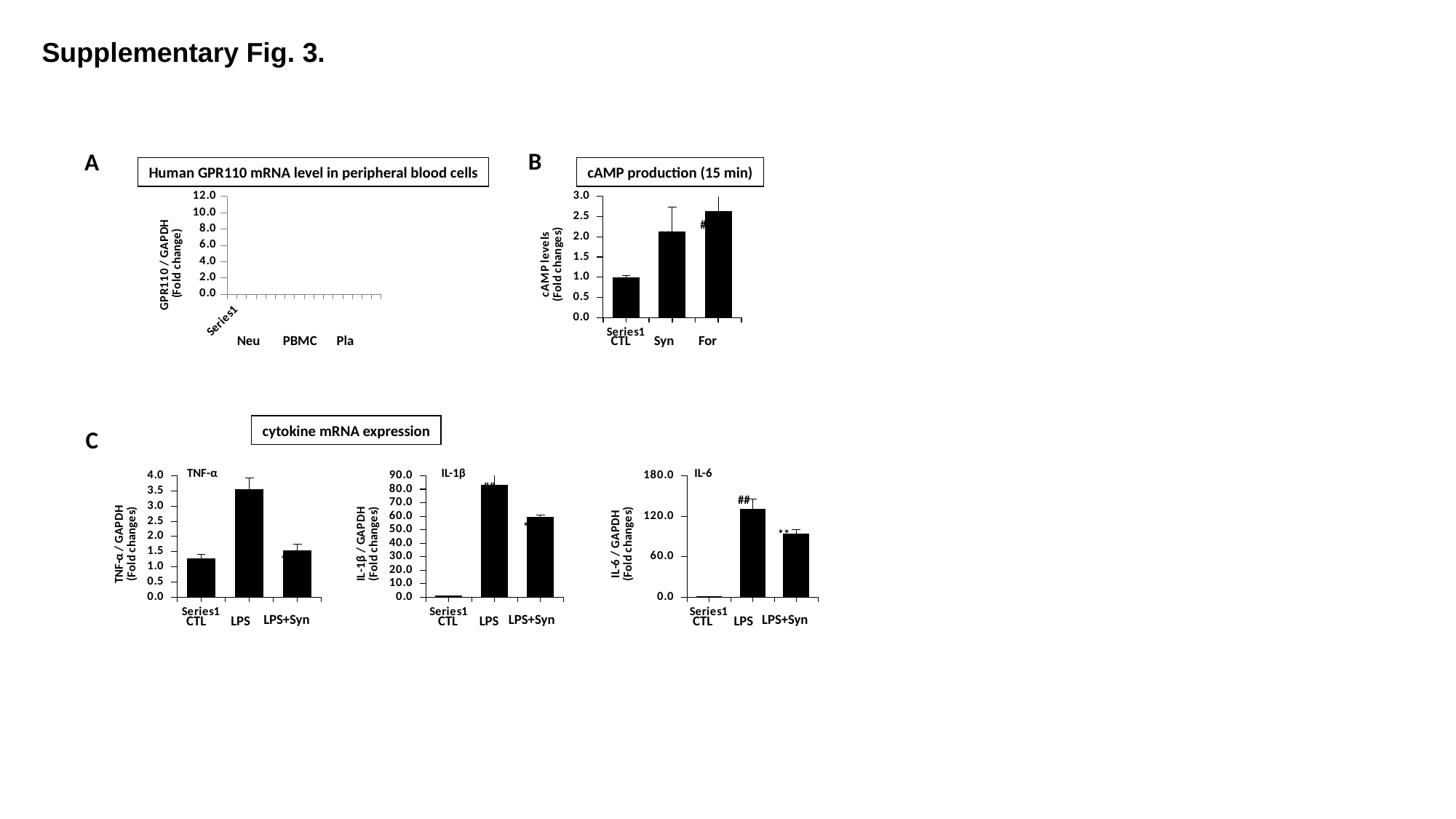

Supplementary Fig. 3.
B
A
Human GPR110 mRNA level in peripheral blood cells
cAMP production (15 min)
### Chart
| Category | |
|---|---|
| | 96.72446558288678 |
| | 1.873539128143185 |
| | 1.0 |
### Chart
| Category | |
|---|---|
| | 1.001198508673461 |
| | 2.138604869073259 |
| | 2.628094444444445 |##
##
Neu
PBMC
Pla
CTL
Syn
For
cytokine mRNA expression
C
TNF-α
IL-1β
IL-6
### Chart
| Category | |
|---|---|
| | 1.279143176232137 |
| | 3.550898748404846 |
| | 1.529934837035491 |
### Chart
| Category | |
|---|---|
| | 1.0 |
| | 83.25558312767635 |
| | 59.4306866980708 |
### Chart
| Category | |
|---|---|
| | 1.0 |
| | 130.429603783261 |
| | 94.5256979361725 |##
##
##
**
**
**
LPS+Syn
LPS+Syn
LPS+Syn
CTL
LPS
CTL
LPS
CTL
LPS
